# Astrocyte-derived tissue Transglutaminase affects fibronectin deposition, but not aggregation, during cuprizone-induced demyelination

**DOI:** 10.1038/srep40995

**Published:** 2017-01-27

**Authors:** Nathaly Espitia Pinzon, Berta Sanz-Morello, John J. P. Brevé, John G. J. M. Bol, Benjamin Drukarch, Jan Bauer, Wia Baron, Anne-Marie van Dam

**Affiliations:** 1 VU University Medical Center, Neuroscience Campus Amsterdam, Dept. Anatomy and Neurosciences, Amsterdam, 1081 HV, The Netherlands; 2 Center for Brain Research, Dept. Neuroimmunology, Vienna, A-1090, Austria; 3 University Medical Center Groningen, Dept. of Cell Biology, Groningen, 9713 AV, The Netherlands

## Abstract

Astrogliosis as seen in Multiple Sclerosis (MS) develops into astroglial scarring, which is beneficial because it seals off the site of central nervous system (CNS) damage. However, astroglial scarring also forms an obstacle that inhibits axon outgrowth and (re)myelination in brain lesions. This is possibly an important cause for incomplete remyelination in the CNS of early stage MS patients and for failure in remyelination when the disease progresses. In this study we address whether under demyelinating conditions *in vivo*, tissue Transglutaminase (TG2), a Ca^2+^ -dependent enzyme that catalyses posttranslational modification of proteins, contributes to extracellular matrix (ECM) deposition and/or aggregation. We used the cuprizone model for de- and remyelination. TG2 immunoreactivity and enzymatic activity time-dependently appeared in astrocytes and ECM, respectively, in the corpus callosum of cuprizone-treated mice. Enhanced presence of soluble monomeric and multimeric fibronectin was detected during demyelination, and fibronectin immunoreactivity was slightly decreased in cuprizone-treated TG2^−/−^ mice. *In vitro* TG2 overexpression in astrocytes coincided with more, while knock-down of TG2 with less fibronectin production. TG2 contributes, at least partly, to fibronectin production, and may play a role in fibronectin deposition during cuprizone-induced demyelination. Our observations are of interest in understanding the functional implications of TG2 during astrogliosis.

Multiple Sclerosis (MS) is a chronic, inflammatory, demyelinating disease of the human central nervous system (CNS) that mainly affects young adults between 20 years and 40 years of age^1^,^2^. MS patients suffer, primarily, from loss of motor and sensory function^3^, visual problems^4^ and cognitive impairment^5^. The pathophysiology of MS consists mainly of inflammation^6^, demyelination^7^ and neurodegeneration^8^,^9^,^10^,^11^ contributing to neurological deficits, which result in progressive disability^12^,^13^. Following tissue injury, several cell types, including microglia and infiltrated macrophages, act as antigen presenting cells, produce inflammatory mediators, and phagocytize myelin and axonal debris^11^. The continuous presence of inflammatory cytokines and oxygen radicals can ultimately compromise neuronal survival and activate local astrocytes^14^,^15^. Upon activation, astrocytes adopt a hypertrophic morphology and increase the expression of e.g. intermediate filament glial fibrillary acidic protein (GFAP), a process known as astrogliosis^16^. During astrogliosis, astrocytes can produce pro-and anti-inflammatory cytokines and chemokines, and moreover elevate the production of extracellular matrix (ECM) proteins^17^,^18^,^19^,^20^. Severe astrogliosis can result in astroglial scarring, which may be beneficial because it seals off the site of CNS damage^21^,^22^. However, the astroglial scar also forms an obstacle to both axon outgrowth and (re)myelination in brain lesions^23^,^24^. This is assumed to be an important cause for the incomplete remyelination in the CNS of early stage MS patients and for the failure of remyelination when the disease progresses^25^,^26^.

Tissue Transglutaminase (TG2) is thus far the best characterized member of a family of Ca^2+^ -dependent enzymes that catalyse posttranslational modification of proteins by cross-linking proteins via ε-(γ-glutamyl)lysine isopeptide bonds or through incorporating primary amines at selected peptide-bound glutamine residues^27^. TG2 is expressed in the cytoplasm, cell organelles or on the surface of a wide variety of cells, and can be deposited into the ECM^28^. Various β-integrins interact with TG2, forming β-integrin-TG2 complexes, on the cell surface facilitating binding of integrins to ECM proteins, like fibronectin^27^,^29^,^30^,^31^. In this manner, TG2 can contribute to cell-matrix interactions, which affect cell spreading and migration important during wound healing^27^. Previously, we have shown that TG2 is present in and on the surface of cultured rat astrocytes and interacts with fibronectin to mediate astrocyte adhesion and migration *in vitro*^32^. In addition, TG2 immunoreactivity was observed in astrocytes in the vicinity of fibronectin in chronic active MS lesions^33^ suggesting a possible role for this enzyme in the formation and/or preservation of the astroglial scar. Indeed, TG2 has been implicated in other pathological scarring conditions, such as pulmonary and liver fibrosis^34^,^35^,^36^. Moreover, a strong association between renal fibrosis, the expression of TG2 and its cross-linking product ε-(γ-glutamyl)lysine isopeptide was not only found in animal models^37^,^38^ but also in human renal biopsy tissue^39^, where it affected cross-linking of the renal ECM, accelerating ECM deposition and conferring resistance to the proteolytic activity of metalloproteinases.

In chronically demyelinated MS lesions, clear deposition of ECM proteins, including fibronectin and laminin has been observed^40^,^41^,^42^. Under these conditions, fibronectin inhibits (re)myelination^42^, whereas laminin is a myelination permissive extracellular matrix molecule^43^,^44^. In the present study we hypothesize, that under demyelinating conditions *in vivo*, TG2 expression and activity is increased and that this enzyme contributes to ECM deposition and/or aggregation. To test our hypothesis, we used the cuprizone model for de-and remyelination. Cuprizone is a copper chelator that is given orally to mice and results in varying degrees of oligodendroglial damage and demyelination in the CNS^45^,^46^. This model might be an appropriate model for studying chronic pathology in MS, since demyelination in this model is accompanied by a strong astrogliosis and microgliosis, as seen in MS patients^47^. This model, however, does not show the disturbance of the blood-brain barrier as seen in MS and is therefore without a profound influx of immune cells^46^,^48^,^49^, comparable to the hypocellular character of chronic, demyelinated MS lesions^50^.

## Results

### Validation of the cuprizone model

**Figure 1:**
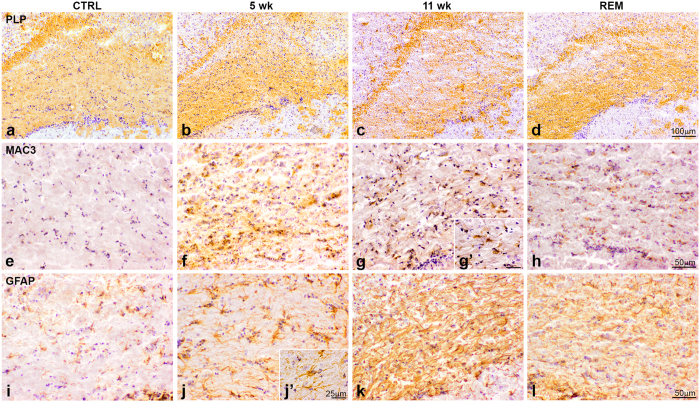
Validation of the cuprizone model. PLP (**a**–**d**), Mac-3 (**e**–**h**) and GFAP (**i**–**l**) immunoreactivity in the corpus callosum of control (CTRL) animals (**a**,**e**,**i**), after 5 (**b**,**f**,**j**) and 11 weeks (**c**,**g**,**k**) of cuprizone treatment (5 wk and 11 wk) and (**d**,**h**,**l**) after 5 weeks of cuprizone treatment followed by 2 weeks of normal chow (REM). Scale bar is 100 μm (**a**–**d**), 50 μm (**e**–**l**) or 25 μm (**g’**,**j’**), n = 4.

The cuprizone model was used to examine the phenotype of astrocytes, ECM composition and the expression of TG2 during de-and remyelination of the corpus callosum. The model was validated by studying de-and remyelination, as well as astrogliosis and microglial activation by myelin proteolipid protein (PLP), GFAP and Mac-3 immunohistochemistry, respectively. In the corpus callosum of control mice clear PLP immunoreactivity was observed (Fig. 1a). However, 5 weeks of cuprizone treatment resulted in apparent demyelination of the corpus callosum (Fig. 1b). Demyelination progressed and extended along this white matter structure in animals treated for 11 weeks with cuprizone (Fig. 1c). Conversely, in animals fed with a cuprizone-supplemented diet for 5 weeks and then fed with normal food for 2 additional weeks, remyelination of the corpus callosum occurred as was apparent by the increase in PLP immunoreactivity in this area (Fig. 1d). The presence of reactive microglia was studied by immunodetection of Mac-3, a membrane glycoprotein expressed by reactive macrophages and microglia^51^. Control animals showed relatively little Mac-3 immunoreactivity in the corpus callosum (Fig. 1e). However, after 5 weeks of cuprizone treatment, the presence of Mac-3 positive microglia in the corpus callosum was evident (Fig. 1f), which was less when animals were treated for 11 weeks with cuprizone (Fig. 1g,g’) and in animals experiencing remyelination during 2 weeks (Fig. 1h). GFAP immunoreactivity was more apparent in animals treated for 5 weeks with cuprizone (Fig. 1j,j’) compared to the control animals (Fig. 1i). Especially after 11 weeks (Fig. 1k) astrocytes expressed more GFAP and formed dense networks of hypertrophic astrocytes, contributing to astroglial scarring. This was not apparent during remyelination, though GFAP immunoreactivity remained evident (Fig. 1l).

### TG2 and fibronectin but not laminin immunoreactivity were elevated during demyelination

**Figure 2:**
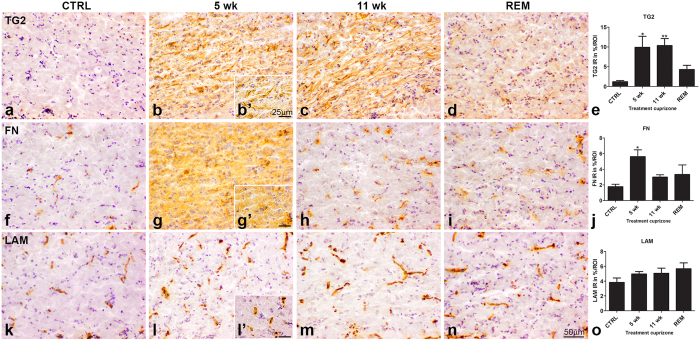
Immunoreactivity of TG2, fibronectin and laminin. TG2 (**a**–**d**), fibronectin (FN) (**f**–**i**) and laminin (LAM) (**k**–**n**) immunoreactivity in the corpus callosum of control (CTRL) animals (**a**,**f**,**k**), after 5 (**b**,**g**,**l**) and 11 weeks (**c**,**h**,**m**) of cuprizone treatment (5 wk and 11 wk) and (**d**,**i**,**n**) after 5 weeks of cuprizone treatment followed by 2 weeks of normal chow (REM). Scale bar is 50 μm (**a**–**d**,**f**–**n**) or 25 μm (**b’**,**g’**,**l’**). Semi-quantitative analysis of the level of immunoreactivity (IR) is quantified in percentage per region of interest (ROI) of the corpus callosum and is presented for TG2 (**e**), FN (**j**) and LAM (**o**). TG2 immunoreactivity is significantly increased after 5 and 11 weeks of cuprizone treatment (p < 0.05 and p < 0.01, respectively) compared to controls (**e**). FN immunoreactivity is significantly increased (p < 0.05) after 5 weeks of cuprizone treatment (**j**). LAM immunoreactivity is statistically unchanged across all groups (**o**). Data are shown as mean + SEM, n = 4, *p < 0.05 and **p < 0.01.

To examine the presence of TG2 in relation to the production and deposition of ECM proteins during de-and remyelination, immunoreactivity of TG2, fibronectin and laminin in the corpus callosum was examined in animals during acute demyelination (after 5 weeks of cuprizone treatment), chronic demyelination (after 11 weeks of cuprizone treatment) and remyelination (5 weeks of cuprizone treatment followed by 2 weeks of normal food). Specificity of the antibody for the TG2 antigen was first established by pre-adsorption of the TG2 antibody with recombinant TG2 from guinea pig liver prior to incubation of the sections. This led to a strong decrease in TG2 immunoreactivity in the corpus callosum of mice treated for 5 weeks with cuprizone (Supplementary Fig. S1), indicating the specificity of the antibody for the TG2 antigen. TG2 immunoreactivity was hardly visible in control mice (Fig. 2a). The limited TG2 immunoreactivity that was found, was confined to the endothelium surrounding blood vessels as shown by others^52^,^53^. After cuprizone treatment, however, TG2 appeared in the brain parenchyma, within cells and in the ECM (Fig. 2b,b’). This was still present following 2 weeks of remyelination (Fig. 2d), although less evident compared to only cuprizone-treated animals. Semi-quantification of TG2 immunoreactivity indicated that the percentage of TG2 immunoreactivity within the indicated ROI was significantly increased in animals after 5 weeks (9.90 ± 2.80%) and 11 weeks (10.36 ± 1.74%) of cuprizone treatment (p < 0.05 and p < 0.01, respectively) compared to controls (1.19 ± 0.27%). This increase was no longer significant during remyelination (4.28 ± 1.08%) (Fig. 2e). Fibronectin immunoreactivity was present in control animals mostly related to blood vessels (Fig. 2f) in line with reports by others^40^,^41^. After 5 weeks of cuprizone treatment, fibronectin immunoreactivity appeared additionally widespread in the corpus callosum, specifically in the ECM (Fig. 2g,g’) and was significantly increased (5.63 ± 0.85%; p < 0.05) compared to control mice (1.78 ± 0.31%) (Fig. 2j). Fibronectin immunoreactivity was still visible and localized in proximity to blood vessels and surrounding ECM in animals after 11 weeks of cuprizone treatment and during remyelination (Fig. 2h,i), but did not reach statistical significance anymore (3.00 ± 0.29% and 3.34 ± 1.22%, respectively) (Fig. 2j). Laminin immunoreactivity in the corpus callosum was located around blood vessels in all groups (Fig. 2k–n) and did not change visibly between the treated groups and control mice. Moreover, semi-quantification indicated that the percentage of laminin immunoreactivity remained statistically unchanged across all groups (Fig. 2o).

### TG2 activity is increased during demyelination

**Figure 3:**
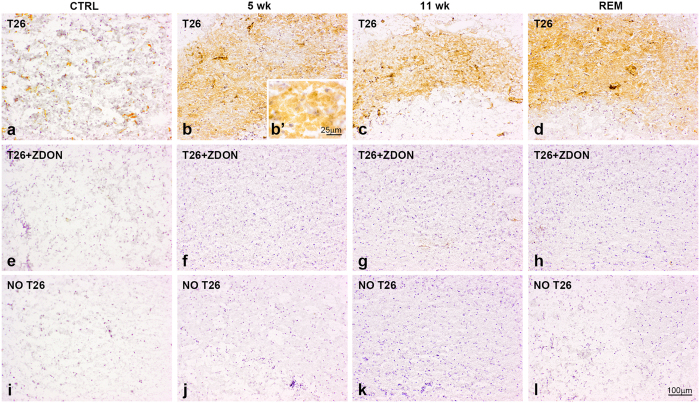
TG2 activity is increased during de- and remyelination. Incorporation of T26 is indicative of TG2 activity in the corpus callosum of control animals (CTRL) (**a**,**e**,**i**), after 5 weeks (5 wk) of cuprizone treatment (**b**,**b’**,**f**,**j**), after 11 weeks of cuprizone (11 wk) (**c**,**g**,**k**) and after 5 weeks of cuprizone treatment followed by 2 weeks of normal chow (REM) (**d**,**h**,**l**). No active TG2 is detected in the corpus callosum of CTRL or cuprizone-treated animals, when sections were pre-incubated with 100 μM Z-DON, a selective inhibitor of TG2 (**e**–**h**) or when sections were incubated without T26 (**i**–**l**). Scale bars are 100 μm (**a**–**l**) or 25 μm (**b’**).

**Figure 4:**
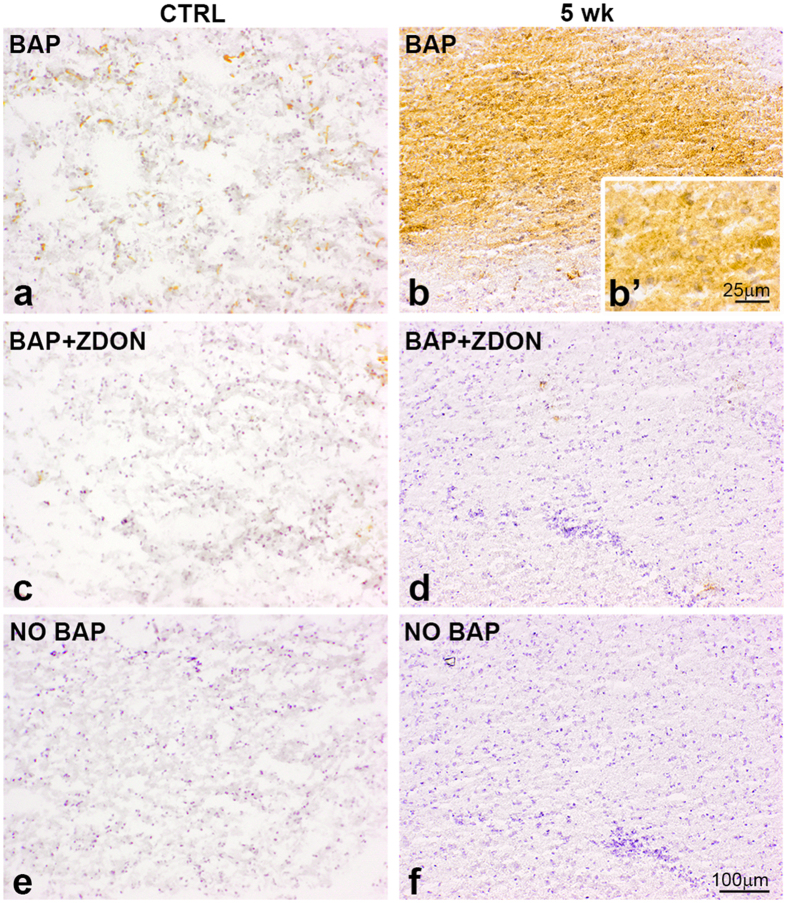
Transglutaminase activity is increased during demyelination. Incorporation of BAP is indicative of transglutaminase activity in the corpus callosum of control animals (CTRL) (**a**,**c**,**e**) and after 5 weeks (5 wk) of cuprizone treatment (**b**,**b’**,**d**,**f**). No active TG2 is detected in the corpus callosum of CTRL or cuprizone-treated animals, when sections were pre-incubated with 100 μM Z-DON, a selective inhibitor of TG2 (**c**,**d**) or when sections were incubated without BAP (**e**,**f**). Scale bars are 100 μm (**a**–**f**) or 25 μm (**b**’).

Using immunohistochemistry we were able to detect elevated levels of TG2 immunoreactivity in the corpus callosum during demyelination. To examine whether this TG2 was enzymatically active, we determined the enzymatic activity of TG2 in the corpus callosum of the different groups of mice by using T26, a specific TG2 substrate, and BAP, a general substrate for transglutaminases. Adding either biotinylated substrate to the sections resulted in little signal in the corpus callosum of control mice (Figs 3a and 4a). Any activity that was seen, was limited to the endothelium surrounding blood vessels as shown by others^54^. After treatment with cuprizone for 5 weeks an increased activity widespread in the corpus callosum was evident and appeared to be located in the ECM (Figs 3b and 4b). This signal disappeared when the sections were co-incubated with the specific TG2 inhibitor Z-DON (Figs 3e,f and 4c,d). These data indicate the presence of increased TG2 activity after 5 weeks of cuprizone treatment. At 11 weeks of cuprizone treatment TG2 activity was still clearly visible (Fig. 3c). Moreover, TG2 activity seemed to be increased during remyelination (Fig. 3d) compared to control as well as groups that received cuprizone for 5 or 11 weeks (Fig. 3a–c). This signal also disappeared in the presence of the TG2 inhibitor Z-DON (Fig. 3g,h). As a technical control, sections from all groups were incubated in the absence of T26 or BAP, resulting in no immunoreactivity (Figs 3i–l and 4e,f).

### TG2 is present in astrocytes during de- and remyelination

**Figure 5:**
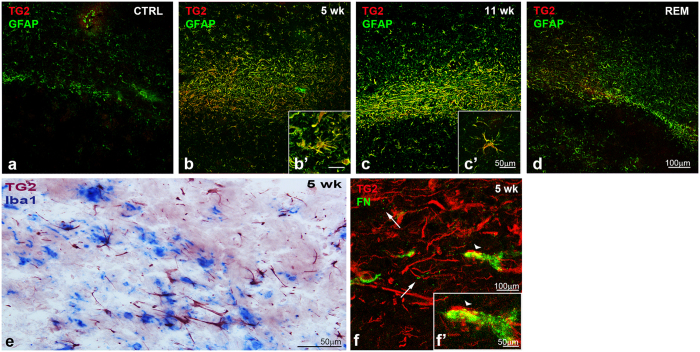
TG2 co-localizes with GFAP and fibronectin but not with Iba1 during demyelination. TG2 and GFAP immunoreactivity (**a**–**d**) in the corpus callosum of control (CTRL) animals (**a**), after 5 (**b**,**b**’) and 11 weeks (**c**,**c**’) of cuprizone treatment (5 wk and 11 wk) and (**d**) after 5 weeks of cuprizone treatment followed by 2 weeks of normal chow (REM) showed that TG2 is present in astrocytes (**b**–**d**) Scale bars are 100 μm (**a**–**d**) or 50 μm (**b’**,**c’**). Consecutive staining of Iba1 and TG2 at 5 weeks (5 wk) of cuprizone treatment showed that TG2 is not present in microglia (**e**). At 5 weeks of cuprizone treatment, fibronectin immunoreactivity (FN) is seen in the parenchyma in a spot-like pattern in close proximity to astrocyte-like cells expressing TG2 (indicated by arrows in **f**). Some astrocyte-like cells also showed co-localization of fibronectin with TG2 (shown by arrowheads in (**f**,**f’**). Scale bars are 50 μm (**e**) and 100 μm (**f**) or 50 μm (**f**’).

Since TG2 and fibronectin immunoreactivity were both increased in the corpus callosum during demyelination, we questioned whether TG2 plays a role in the interaction of specific cells with fibronectin. Co-localization of TG2 with GFAP, Iba1 or fibronectin was therefore examined in the corpus callosum. TG2 and GFAP showed extensive co-localization after 5 (Fig. 5b,b’) and 11 weeks of cuprizone treatment (Fig. 5c,c’). This was still visible after remyelination (Fig. 5d), indicating that astrocytes are the main cells to express TG2 during de-and remyelination. Additionally, consecutive staining of Iba1 and TG2 at 5 weeks of cuprizone treatment showed that TG2 is not present in microglia (Fig. 5e). At 5 weeks of cuprizone treatment, fibronectin immunoreactivity was seen in the parenchyma in a spot-like pattern in close proximity to cells with an astrocyte-like morphology. These cells also showed some co-localization of fibronectin with TG2 (shown by arrows and arrowheads respectively in Fig. 5f,f’).

### Fibronectin mono-and multimers during de-and remyelination

**Figure 6:**
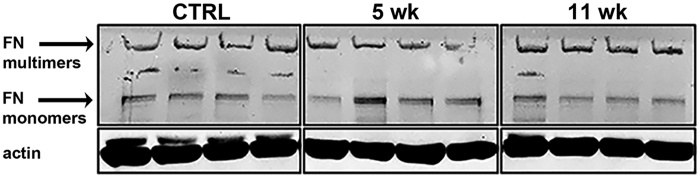
Fibronectin monomers and multimers are affected by cuprizone treatment. Deoxycholate-soluble fibronectin (FN) monomers of 220 kDa show a slight increase after 5 weeks (5 wk) weeks of cuprizone treatment, compared to control (CTRL) animals. Deoxycholate-soluble fibronectin multimers were confined to the loading wells due their size and also appear to be slightly increased after 11 weeks (11 wk) of cuprizone treatment. Samples were derived from the same experiment and blots were processed in parallel. Cropping was used in the figure. Full-length blots are presented in Supplementary Fig. 2.

Fibronectin is normally found as a disulphide-bound dimer with two subunits of 220–250 kDa each, but can assemble into a complex network of high molecular weight aggregates, which are insoluble in the detergent deoxycholate (DOC)^55^,^56^. To examine whether fibronectin formed aggregates in the cuprizone model, DOC soluble and DOC insoluble monomeric and multimeric isoforms of fibronectin were examined in tissue punches of the corpus callosum by western blotting. The DOC-insoluble fraction of total proteins from the corpus callosum did not show any signal (data not shown), suggesting an absence of DOC-insoluble fibronectin mono- and multimers in the ECM. The DOC-soluble fraction however, showed a slight increase in fibronectin dimers, which were reduced by western blotting conditions and appeared as monomers of 220 kDa after 5 weeks of cuprizone treatment (Fig. 6). DOC-soluble fibronectin multimers were confined to the loading wells due to their size. The amount of fibronectin multimers seemed to be slightly increased after 11 weeks of cuprizone treatment (Fig. 6).

### Fibronectin immunoreactivity is reduced in cuprizone-treated TG2^−/−^ mice

**Figure 7:**
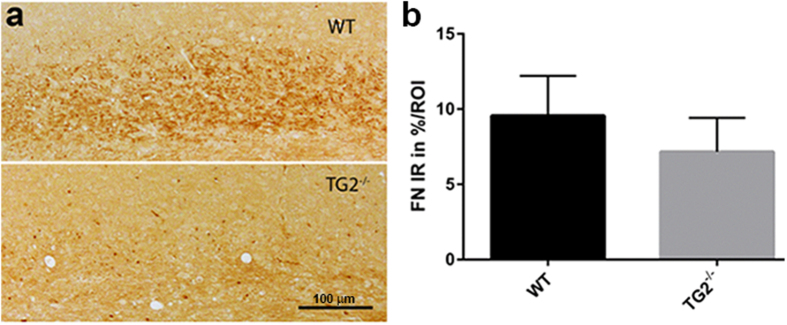
Fibronectin immunoreactivity in cuprizone-treated TG2^−/−^ and wildtype mice. Fibronectin immunoreactivity in the corpus callosum of TG2^−/−^ and wildtype mice treated with cuprizone for 5 weeks (**a**). Scale bar is 100 μm. The level of fibronectin immunoreactivity (IR) is semi-quantified and expressed as percentage of IR per region of interest (ROI) in the corpus callosum (**b**). Fibronectin immunoreactivity is less present in TG2^−/−^ mice compared to littermate wildtype mice, but did not reach statistical significance. Data are shown as mean + SEM, n = 6.

To determine whether TG2 contributes to the increase in fibronectin immunoreactivity (as shown in Fig. 2f,g), TG2^−/−^ and littermate control mice were treated for 5 weeks with cuprizone. In TG2^−/−^ mice less fibronectin immunoreactivity (7.16 ± 2.26%) was observed in the corpus callosum compared to wildtype mice (9.56 ± 2.64%; Fig. 7), although it did not reach statistical significance.

### Fibronectin expression is affected in astrocytes by up-and downregulation of TG2

**Figure 8:**
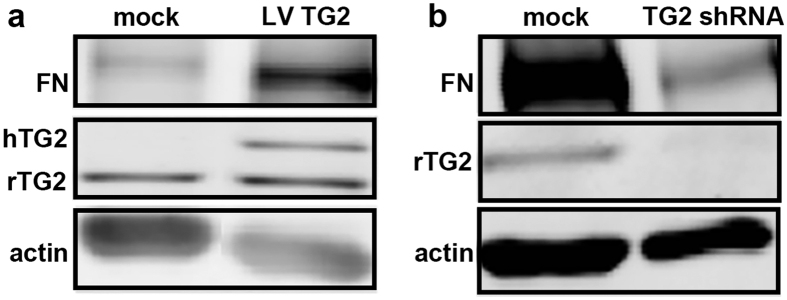
Fibronectin expression is affected in astrocytes after up- and downregulation of TG2. Lentiviral overexpression of TG2 (LV TG2) in primary rat astrocytes results in an extra band for TG2 (hTG2) on western blot, indicative of the human TG2 which is slightly bigger compared to the endogenous rat TG2 (rTG2; 78 kDa). Fibronectin expression (220 kDa) is increased in primary rat astrocytes after lentiviral upregulation of TG2 (**a**). Alternatively, downregulation of TG2 (TG2 shRNA) decreases fibronectin expression (**b**). Blots of *in vitro* upregulation (**a**) and downregulation (**b**) of TG2 were processed in parallel. Cropping was used in the figure. Full-length blots are presented in Supplementary Fig. 3.

To determine whether TG2 can directly affect fibronectin production, we studied fibronectin production of astrocytes overexpressing TG2. The overexpressed human TG2 was visible by an extra band, slightly higher than endogenous rat TG2 (Fig. 8a). Moreover, fibronectin expression was elevated (Fig. 8a) compared to the mock transduced control. Alternatively, fibronectin expression was decreased when TG2 was downregulated (Fig. 8b).

## Discussion

In the present study we showed that during demyelination, TG2 immunoreactivity and enzymatic activity time-dependently appear in astrocytes and ECM in the corpus callosum of mice treated with cuprizone. Similarly, enhanced presence of monomeric and multimeric fibronectin is detected during demyelination. Subsequent *in vitro* studies suggest that enhanced TG2 expression in astrocytes coincides with more astrocytic fibronectin, and knock-down of TG2 decreases fibronectin production. These data suggest that TG2 directly contributes to fibronectin production, and may play a role in fibronectin deposition during cuprizone-induced demyelination.

The cuprizone model as used in the present study has been widely used before to determine which cells and factors contribute to demyelination and remyelination processes as observed in MS^48^,^57^. We observed clear demyelination during cuprizone treatment which resulted in remyelination upon ablation of the cuprizone diet. Moreover, an expected early increase in Mac-3 expression in microglia was seen which was reduced upon longer exposure to cuprizone. Finally, an elevation in GFAP expression was found during demyelination, and reduced slightly during remyelination. These time-dependent changes in cellular responses found during demyelination and remyelination were similar to those reported by others^58^,^59^,^60^. In this model, TG2 immunoreactivity was enhanced in particular during demyelination, and present in astrocytes. What induces the increase in TG2 production in astrocytes during cuprizone-induced demyelination is not known, but inflammatory mediators and glutamate have been shown to elevate TG2 expression in astrocytes^32^,^61^,^62^. The activity of this enzyme was clearly present and mostly located in the ECM. This is in line with observations that intracellular TG2 is usually inactive, but becomes activated extracellularly when redox conditions are altered^63^,^64^. Enhanced oxidative stress is clearly described in the cuprizone model^65^,^66^,^67^. In addition, glutamate excitotoxicity is enhanced during cuprizone-induced demyelination^68^. Thus, elevated presence and activity of TG2 is likely to play a role in the cuprizone model.

Simultaneous to increased TG2 immunoreactivity, also fibronectin, but not laminin, immunoreactivity increasingly appeared in the ECM during demyelination. DOC-insoluble fibronectin aggregates are considered to disturb remyelination^42^. We observed a slight increase in DOC-soluble fibronectin mono- and multimers during demyelination, but did not find conclusive fibronectin aggregates. It has been suggested before that inflammation, i.e., damage to the blood-brain barrier is essential to obtain real fibrosis, which is absent in the cuprizone model^59^,^69^,^70^. This might also explain why fibronectin aggregates were previously found at the relapse phase in chronic relapsing EAE and multiple sclerosis lesions, but not in lysolecithin-induced demyelination^42^. Our observations, that fibronectin multimers are slightly more present after chronic demyelination (11 weeks of cuprizone treatment), may suggest a pre-aggregation state.

Subsequent analysis of TG2^−/−^ mice after 5 weeks of cuprizone administration showed slightly less fibronectin immunoreactivity in the demyelinated corpus callosum compared to littermate wildtype mice. This is in line with our *in vitro* studies showing that overexpression of human TG2 in rat astrocytes resulted in more astrocytic fibronectin production, while in astrocytes with downregulated TG2, the amount of astrocytic fibronectin decreased compared to the mock condition. These data suggest that TG2, at least partly, is involved in fibronectin production and may contribute to fibronectin deposition. This would fit with the observed enhanced amount of extracellular fibronectin when TG2 immunoreactivity is elevated during demyelination.

Also in chronic MS lesions, TG2 immunoreactivity was elevated in astrocytes and observed in close vicinity to fibronectin^33^. Cuprizone-induced demyelination shows a clear increase in TG2 expression in astrocytes and extracellular fibronectin as in chronic MS lesions. However, in contrast to MS lesions, no fibronectin aggregates can be observed as in MS lesions when remyelination fails^40^,^41^,^71^, suggesting that additional, likely inflammatory factors are necessary in the formation of DOC-insoluble fibronectin aggregates. Actually, in the cuprizone model ablation of cuprizone results in remyelination which favours the absence of aggregated ECM proteins. Moreover, we previously observed that TG2 contributes to remyelination in the cuprizone model, which is probably due to TG2 promoting differentiation of oligodendrocyte precursor cells^45^. Thus, TG2 may have cell-specific effects, depending on the timing of its presence during de- and re-myelination processes.

In conclusion, both TG2 immunoreactivity in astrocytes and TG2 enzymatic activity in the ECM were increased during cuprizone-induced demyelination, which coincided with an increase in fibronectin deposition, but not aggregation. Fibronectin production was reduced in TG2^−/−^ mice and TG2 downregulated astrocytes *in vitro*. Moreover, overexpression of TG2 enhanced fibronectin production by astrocytes *in vitro*. Our novel observations on TG2 in this model of de-and remyelination are of interest in better understanding the possible involvement of astrocyte-derived TG2 in the pathogenesis of fibronectin deposition during astrogliosis in MS.

## Methods

### Cuprizone model

Demyelination and remyelination was induced in 8-week old C57BL/6 mice (Charles River Laboratories, Portage, MI). To this end, the animals (n = 4/group) were fed ad libitum with normal powdered chow (control group, Abdiets, Woerden, the Netherlands), or with powdered chow to which 0.2% (w/w) cuprizone (bis-cyclohexanone oxaldihydrazone, Sigma-Aldrich Inc., St. Louis, Missouri, USA) was added during 5 or 11 weeks respectively. Another group of mice was fed with 0.2% w/w cuprizone for 5 weeks and subsequently fed with normal food for 2 more weeks to examine remyelination. Immediately after the end of the treatment, animals were sacrificed by decapitation. The control group was sacrificed after 5 weeks. Their brains were extracted, hemispheres were separated and each directly frozen in liquid nitrogen and subsequently stored at −80 °C. The same cuprizone treatment was given to 8–10 week old C57Bl/6 TG2^−/−^ (n = 6) mice^72^ that were backcrossed for at least 12 generations, and to littermate TG2^+/+^ genotype control (n = 6) mice which were kept in individual ventilated cages with food and water accessible ad libitum. Both groups were sacrificed after 5 weeks of cuprizone treatment, their brains extracted, fixed overnight in 4% PFA in 0.2 M phosphate buffer and subsequently embedded in paraffin. These studies were approved by the VU University Medical Center committee on Animal Experimentation (approval numbers ANW 13–02 and DMF 101122), and carried out in strict accordance with their guidelines.

### Immunohistochemistry on cryosections

Coronal cryosections (10 μm) at the level of the corpus callosum from fresh frozen single hemispheres were cut from cuprizone-treated and control mice, dried and stored at −80 °C until use. Sections were allowed to dry for 2 h at room temperature (RT), and fixed with 4% paraformaldehyde (PFA) in 0.2 M phosphate buffered saline (PBS, pH 7.4) for 20 minutes (min) at RT. Sections to be stained for PLP were additionally dehydrated in ethanol 70%, 90%, 96%, and 100% (3 times) to remove fatty substances from the myelin, and then rehydrated in 96% ethanol, 90% ethanol, 70% ethanol, demineralized water and Tris-buffered saline (TBS, pH 7.4). In all sections, endogenous peroxidase activity was inhibited by incubation for 15 min at RT with 0.3% hydrogen peroxidase and 0.1% sodium azide in TBS. After 3 washes in TBS, non-specific binding sites were blocked by incubating the sections for 20 min at RT in 5% non-fat dried milk or 3% bovine serum albumin (BSA, Sigma-Aldrich; Mac-3 staining) in TBS containing 0.5% Triton X-100 (TBS-T; pH 7.6) Alternatively, sections to be stained for PLP were pre-treated with IHS mouse-to-mouse ISO blocking solution (ImmunoLogic, Duiven, the Netherlands). Subsequently, all sections were incubated overnight in a humid chamber with one of the primary antisera (see Table 1 for details) diluted in their corresponding blocking solution at 4 °C. Thereafter, sections were washed in TBS and incubated in a humid chamber for 2 hr at RT with their corresponding biotinylated goat anti-mouse IgG’s, goat anti-rabbit IgG’s, goat anti-rat IgG’s or donkey anti-goat IgG’s (1:400; Jackson ImmunoResearch Laboratories Inc., West Grove, Pennsylvania, USA) in TBS-T. Sections were washed in TBS and incubated with horse radish peroxidase (HRP)-labelled avidin-biotin complex (ABC complex, 1:400; Vector Laboratories, Burlingame, CA, USA) in TBS-T for 1 hr at RT. Sections were washed twice with TBS and once with Tris-HCl. Peroxidase activity was visualized using 3,3-diaminobenzidine (DAB, Sigma, St. Louis, USA) as a chromogen. Finally, sections were counterstained with haematoxylin, dehydrated in graded ethanol solutions, cleared in xylene and coverslipped with Entellan (Merck, Darmstadt, Germany).

For double labelling of Iba1 and TG2, the immunohistochemical procedure was performed by sequential staining of Iba1 and TG2. After incubation with Iba1 (1:2000, Wako Chemicals, see Table 1 for more details), the sections were incubated in a humid chamber for 1 hr at RT with anti-rabbit IgG’s (ImmPRESS™- Alkaline Phosphatase reagent, MP-5402, Vector Laboratories, undiluted). Sections were then washed in TBS and incubated with Vector Blue Alkaline Phosphatase Substrate (SK-5300, Vector Laboratories, undiluted) to visualize Iba1 staining. Subsequently, the sections were washed in TBS and in ethylenediaminetetraacetic acid buffer (EDTA, pH = 9.0). Subsequent incubation of the sections in EDTA buffer for 30 min in a steaming device (MultiGourmet FS 20; Braun, Kronberg/Taunus, Germany) eliminated the bound antibodies without affecting the precipitate. Sections were allowed to regain room temperature (RT), washed three times in TBS and stained for TG2 by an overnight incubation in a humid chamber with the primary antiserum (1:4000, Upstate, see Table 1 for more details) at 4 °C. Thereafter, sections were washed in TBS and incubated in a humid chamber for 2 hr at RT with biotinylated donkey anti-goat IgG’s (1:400; Jackson ImmunoResearch Laboratories Inc., West Grove, Pennsylvania, USA) in TBS-T. Sections were washed in TBS and incubated with horse radish peroxidase (HRP)-labelled avidin-biotin complex (ABC complex, 1:400; Vector Laboratories) in TBS-T for 1 hr at RT. Sections were washed with TBS and peroxidase activity was visualized using VIP Peroxidase Substrate (SK-4600, Vector Laboratories, undiluted) as a chromogen. Finally, sections were washed with TBS and water, dehydrated in graded ethanol solutions, cleared in xylene-replacement (Sigma) and coverslipped with Vectamount (Vector Laboratories).

Images were taken with an Olympus-VANOX-T light microscope. Specificity of TG2 immunoreactivity was confirmed by pre-adsorption of the diluted goat anti-TG2 antibody with recombinant TG2 from guinea pig liver (1 mg/ml, Sigma-Aldrich) in TBS-T, for 1 h at RT on a shaker. This was followed by an overnight incubation at 4 °C before addition of the mixture to the sections, after which the described immunohistochemical procedure was followed.

### Immunohistochemistry of paraffin sections

Paraffin-embedded brains of cuprizone-treated TG2^−/−^ mice and littermate controls were cut in 5 μm sections at the level of the corpus callosum. Sections were deparaffinised in xylene and transferred to distilled water via degrading 90%, 70% and 50% ethanol series. As a pre-treatment, sections were incubated with 20 μg/ml proteinase K (Sigma P2308) in TBS containing 0.02 M CaCl_2_ for 15 min at 37 °C. All further incubation steps were performed in DAKO washing buffer (Dako, Denmark) with additional 10% fetal calf serum. The sections were incubated overnight at 4 °C with a goat-anti-human fibronectin antibody (1:50; Santa Cruz, C-20). Subsequently, sections were incubated for 1 hr at room temperature with biotinylated donkey anti-goat IgG’s (1:200; Jackson ImmunoResearch Laboratories Inc). Sections were washed in TBS and incubated with horse radish peroxidase (HRP)-labelled avidin (1:100; Sigma) for 1 hr at RT. Finally, peroxidase activity was visualized using 3,3-diaminobenzidine (DAB, Sigma, St. Louis, USA) as a chromogen.

### Semi-quantification of immunoreactivity

Semi-quantitative analysis of TG2, fibronectin and laminin immunoreactivity (IR) was performed by measuring the amount of IR per region of interest (ROI, 0.1 mm^2^) in similar areas of the corpus callosum (approximately at Bregma 0.7 mm) ventral from the cingulum. To this end, pictures were taken at a 20 × 3.3 magnification and a standardized threshold procedure was used that distinguished background from specific IR as described by us previously^73^. Subsequently, the amount of specific IR was measured within the defined ROI, and expressed as percentages of TG2, fibronectin and laminin IR per ROI. Data were averaged per treatment group and expressed as mean + SEM. The ROI was kept constant between animals and groups to perform the analysis in a reproducible manner and to be able to compare the amount of TG2, fibronectin and laminin IR between the treatment groups. These semi-quantitative analyses were performed unbiased using CellF Olympus Soft Imaging Solutions GmbH software, version 3.1 (Tokyo, Japan).

### TG2 activity assay

Frozen brain sections (10 μm) were dried for 2 h and then pre-incubated for 20 min at RT with an assay buffer (0.1 M Tris-HCl, 5 mM CaCl_2_, 1 mM dithiothreitol (DTT), 0.1% dimethyl sulfoxide (DMSO)) in the presence of absence of a selective TG2 inhibitor (Z-DON, 100 μM, Zedira)^74^. Subsequently, sections were incubated for 30 min at 37 °C with 50 mM pentylamine-Biotin (BAP, Thermo Scientific), a biotinylated amine TG substrate, or with 50 mM T26 (Covalab, Villeurbanne, France), a specific, biotinylated TG2 substrate, diluted in the assay buffer^75^. After several washes in TBS and water, sections were fixed with 4% PFA in PBS for 20 min at RT. Subsequently, the sections were treated as described for immunohistochemistry. After incubation with horse radish peroxidase (HRP)-labelled avidin-biotin complex, peroxidase activity was visualized using DAB as a chromogen. Images were taken using an Olympus-VANOX-T light microscope. Omission of BAP and T26 substrates served as negative controls.

### Immunofluorescence

For double labelling of TG2 with the astrocyte marker GFAP or with fibronectin, coronal frozen brain sections (10 μm) were dried for 2 h at RT and then fixed with 4% PFA in PBS for 20 min (for TG2/GFAP) at RT. After blocking non-specific antibody binding with 5% non-fat dried milk for 20 min at RT, sections were incubated overnight in a humid chamber with the primary antibodies goat anti-TG2 (1:8000, Upstate) combined with rabbit anti-GFAP (1:4000, DAKO) or with rabbit anti-fibronectin (1:200, Sigma) diluted in blocking buffer at 4 °C (see Table 1 for more details). Subsequently, sections were washed in TBS and incubated in a dark, humid chamber with the appropriate fluorescently labelled IgG’s, i.e. donkey anti-goat IgG Alexa Fluor 594 (1:400, Molecular Probes) combined with donkey anti-rabbit IgG Alexa Fluor 488 (1:400, Molecular Probes) diluted in TBS-T for 2 h at RT. After several washes in TBS, sections were mounted with Vectashield (Vector laboratories Inc.; GFAP/TG2) or with polyvinyl alcohol mounting medium with DABCO (Sigma-Aldrich). Immunofluorescence was examined using a Leica confocal laser scanning microscope (Leica TSC-SP2-AOBS; Leica Microsystems, Wetzlar, Germany).

### Primary rat astrocyte cultures

Primary astrocytes were collected from 1 to 3-day-old Wistar rats (Harlan, Horst, The Netherlands). The rats were decapitated, cortical hemispheres were collected and meninges were removed. Tissue was digested in MEM (Sigma, St. Louis, MO) containing papain (30 U/ml, Sigma), cysteine (0.24 mg/ml; Sigma) and DNase I type IV (40 μg/ml; Sigma). Supernatant was then removed, 1 mL of ovomucoid trypsin inhibitor solution (containing 1 mg/ml trypsin inhibitor; Roche, The Netherlands), 50 μg/ml bovine serum albumin (BSA), and 40 μg/mL DNase was added. Cells were centrifuged 5 minutes at 1000 rpm, and the cells were plated on poly-L-lysine (PLL, 5 μg/ml, Sigma) coated tissue 75 cm^2^ cell culture flasks (Nalge Nunc, Naperville, IL) at a density of 1.5 brains per flask. Cells were cultured in DMEM (Life Technologies, Carlsbad, CA) supplemented with 10% fetal calf serum (FCS; Bodinco, Alkmaar, The Netherlands). Medium was changed twice a week. After two weeks, flasks were shaken at 150 rpm for 1 hour at 37 °C on an orbital shaker to remove microglia. Subsequently, flasks were shaken again at 240 rpm overnight at 37 °C to remove oligodendrocyte precursor cells. The remaining astrocyte monolayer was removed with trypsin. Astrocytes were cultured in T162 flasks (Corning Costar, Lowell, MA) in DMEM supplemented with heat-inactivated 10% FCS.

### Lentiviral modification of TG2

Human wild-type TG2 c-DNA (gift from prof. K. Mehta, University of Texas, M.D. Anderson Cancer Center, Houston, USA) was cloned into the lentiviral vector pCDH (System Bioscience, USA). Mock-transduced cells were used as a control. Primary rat astrocytes were transduced before the shake-off by exposing the cells overnight at 37 °C to lentiviral particles and polybrene (8 μg/ml; Sigma), which facilitated viral particle entry. The cells were then left to recover in DMEM supplemented with 10% FCS, L-glutamine, penicillin and streptomycin for 48 hours, subjected to the shake-off procedure, followed by puromycin selection (1 μg/ml, Sigma) for at least 5 days. Alternatively, TG2 was down-regulated in primary rat astrocytes by lentiviral transduction with TG2 specific shRNA. Therefore, astrocytes were plated on a poly-L-lysine (0.8 μg/cm^2^; Sigma-Aldrich) coated 12 well plate (0.2 × 10^6^ cells/well). The following day, 0.2 × 10^6^ infectious units of virus (IFU) of lentiviral particles (Santa Cruz, sc-270266-V for the rat TG2 shRNA, or sc-108080 for the control shRNA data not shown) was added to the cells. The medium was changed the next day and the same amount of lentiviral particles was added to the cells. The medium was changed again the following day and this was done twice a week, from that point onward by medium containing 2 μg/ml puromycine (Sigma-Aldrich).

### Protein isolation

The remaining hemispheres of the fresh frozen brains from control and cuprizone treated mice were used to examine ECM protein expression and aggregation in the corpus callosum by western blotting. Tissue punches (1 mm diameter) including the corpus callosum area, ventral from the cingulum, were taken from coronal brain sections (20 μm). A total of 20 punches per hemisphere was collected in 90 μl of 2% DOC containing 2 mM EDTA, 5 mM Tris-HCL, 0.1 mM PMSF, 7.5 μM pepstatin-A, 10 μM leupeptin and 0.75 μM aprotinin (all from Sigma-Aldrich). Additionally, primary rat astrocytes were washed three times with PBS (pH 7.4), harvested by scraping, and centrifuged for 7 minutes at 7000 rpm at RT. Pellets were lysed in TNE lysis buffer (50 mM Tris-HCl, 5 mM EDTA, 150 mM NaCl, 1% Triton X-100, and protease inhibitor cocktail; Complete Mini; Roche Diagnostics GmbH), pH 7.4). Protein concentrations of the tissue and cell samples were determined by the Bradford Concentration Assay (BCA; Pierce Biotechnology, Etten-Leur, The Netherlands) as described in the instructions provided with the assay.

### Western blot analysis

Tissue samples for western blotting were centrifuged at 14,000 rpm for 30 min at 4 °C, to separate the DOC-soluble from the DOC-insoluble fraction of total proteins isolated from the corpus callosum. To either tissue fraction 50 mM DTT was added and samples were heated for 10 min at 95 °C. Cellular samples from rat astrocytes in culture were diluted with reducing sample buffer, containing beta-mercaptoethanol, and heated for 5 min at 98 °C. Equal amounts of protein (20 μg) were subjected to 8% SDS-polyacrylamide gel electrophoresis (SDS-PAGE) and subsequently transferred to a nitrocellulose membrane (Li-Cor Biosciences, Lincoln, Nebraska, USA). Membranes were blocked at RT with Odyssey blocking buffer (Li-Cor Biosciences) diluted 1:1 with TBS. Membranes were incubated overnight at 4 °C with rabbit anti-fibronectin (Neomarkers, 1:1000), mouse anti-TG2 (ab2386, Abcam, 1:2000) or mouse anti-β-actin (Abcam, 1:50000). After several washes with TBS containing 0.1% Triton, blots were incubated for 2 h at RT with corresponding goat anti-rabbit IRDye 680LT IgG’s (1:10000, Li-Cor Biosciences), washed in TBS containing 0.1% Triton, and were subsequently incubated for 1 h with donkey anti-mouse IRDye 800CW IgG’s (1:10000, Li-Cor Biosciences) for subsequent antigen detection. All antibody solutions were prepared in the Odyssey blocking buffer (Li-Cor Biosciences) diluted 1:1 with TBS-0.1% Triton. After washing, membranes were scanned for fluorescence emission at 700 and 800 nm, using an Odyssey infrared imaging system (Li-Cor Biosciences). Bands were visualized and their signal intensities were measured using the Odyssey Sa Infrared imaging system (Li-cor Biosciences).

### Statistical analysis

Normal distribution of the data was tested using the Shapiro-Wilk procedure. When the data were normally distributed, a student’s t-test for 2 group comparisons (semi-quantitative analysis of fibronectin immunoreactivity in TG2^−/−^ and wildtype cuprizone treated mice) or one-way analysis of variance (ANOVA) was performed followed by Dunnett’s post hoc test for multiple comparisons compared to the control groups (semi-quantitative analysis of TG2, fibronectin and laminin immunoreactivity in cuprizone treated wildtype mice) by using IBM SPSS software, version 20.0 (IBM Corp, NY, USA). P < 0.05 was considered to represent statistically significant differences.

## Additional Information

**How to cite this article**: Espitia Pinzon, N. *et al*. Astrocyte-derived tissue Transglutaminase affects fibronectin deposition, but not aggregation, during cuprizone-induced demyelination. *Sci. Rep.* **7**, 40995; doi: 10.1038/srep40995 (2017).

**Publisher's note:** Springer Nature remains neutral with regard to jurisdictional claims in published maps and institutional affiliations.

## Supplementary Material

Supplementary Information

## Figures and Tables

**Table 1:** Primary antibodies used for immunohistochemistry.

Antigen	Host species	Final dilution	Source
TG2	Goat	1:8000/1:4000 after staining for Iba1	Upstate, 06-471
Fibronectin	Rabbit	1:200	Sigma-Aldrich, F3648
Fibronectin	Goat	1:50	Santa Cruz (C-20), sc-6952
Laminin	Rabbit	1:600	Abcam, ab7463
GFAP (astrocytes)	Rabbit	1:4000	DAKO, Z0334
Mac-3 (microglia)	Rat	1:200	BD Transduction Labs, 550292
Iba1 (microglia)	Rabbit	1:2000	Wako Chemicals, 019-19741
PLP (myelin)	Mouse	1:500	Serotec, MCA839G
