# Supplementary material for: Astrocyte-derived tissue Transglutaminase affects fibronectin deposition, but not aggregation, during cuprizone-induced demyelination
Source: Sci Rep. 2017 Jan 27;7:40995. doi: 10.1038/srep40995 (PMC5269585; doi:10.1038/srep40995)
Supplement: Supplementary Information [file srep40995-s1.pdf]

Supplemental information

Astrocyte-derived tissue Transglutaminase affects fibronectin deposition, but not aggregation, during cuprizone-induced demyelination

Nathaly Espitia Pinzon[1], Berta Sanz-Morello[1,†], John J. P. Brevé[1], John G. J. M. Bol[1], Benjamin Drukarch[1], Jan Bauer[2], Wia Baron[3#], Anne-Marie van Dam[1,#,*]

[1]VU University Medical Center, Neuroscience Campus Amsterdam, Dept. Anatomy and Neurosciences, Amsterdam, 1081 HV, The Netherlands

[2]Center for Brain Research, Dept. Neuroimmunology, Vienna, A-1090, Austria

[3]University Medical Center Groningen, Dept. of Cell Biology, Groningen, 9713 AV, The Netherlands

[†]present address: Biotech Research and Innovation Center, University of Copenhagen, Copenhagen, 2200, Denmark

[#]shared senior authorship

[*]corresponding author

Email amw.vandam@vumc.nl

The following pages include:

Figure S1

Figure S2

Figure S3

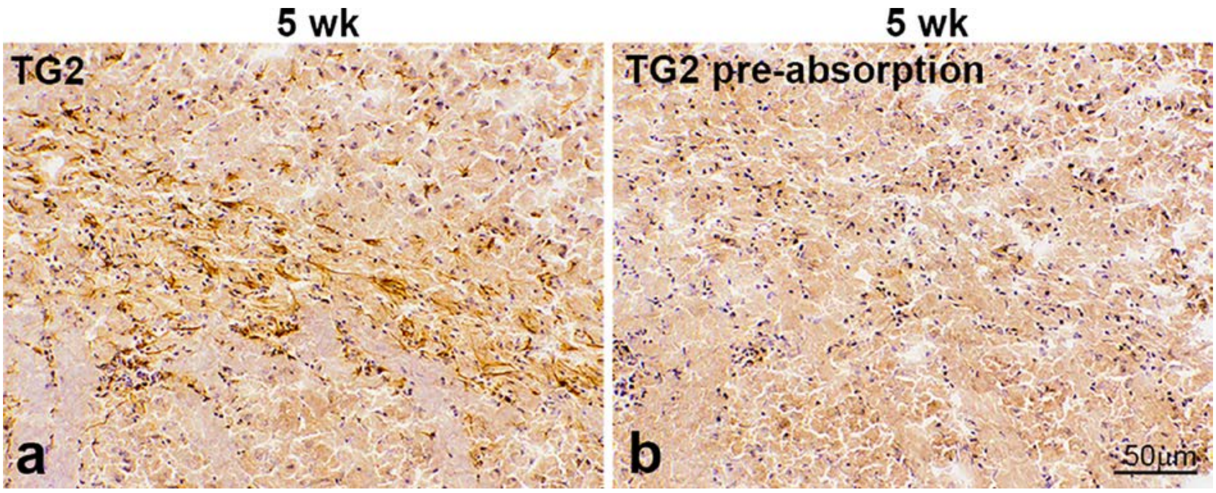

Figure S1. Specificity of TG2 immunoreactivity. TG2 immunoreactivity (a) is seen in the corpus callosum after 5 weeks (5 wk) of cuprizone treatment. Specificity of TG2 stainings is confirmed by pre-adsorption of recombinant TG2 from guinea pig liver with the primary TG2 antibody prior to incubation with the appropriate secondary antibody. After preadsorption with guinea pig TG2 no immunohistochemical signal is seen for the TG2 antibody, demonstrating its specificity for the TG2 antigen (b). Scale bar is 50 µm.

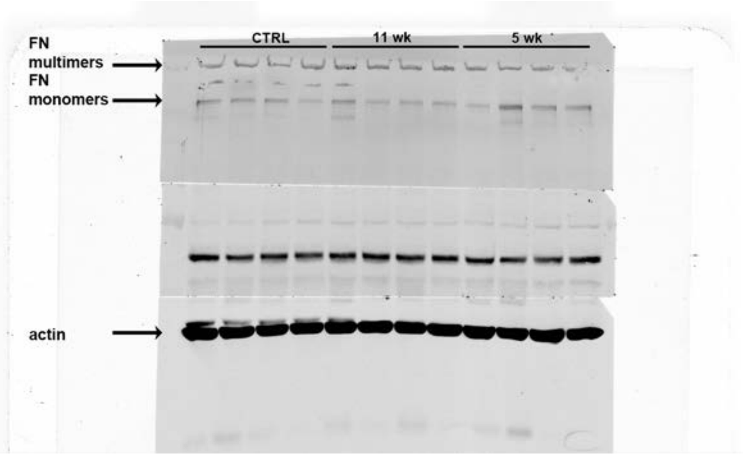

Figure S2. Full-length blot for figure 6. Sample order was changed to CRTL, 5 weeks (wk) and 11 wk of treatment in cropped western blot.

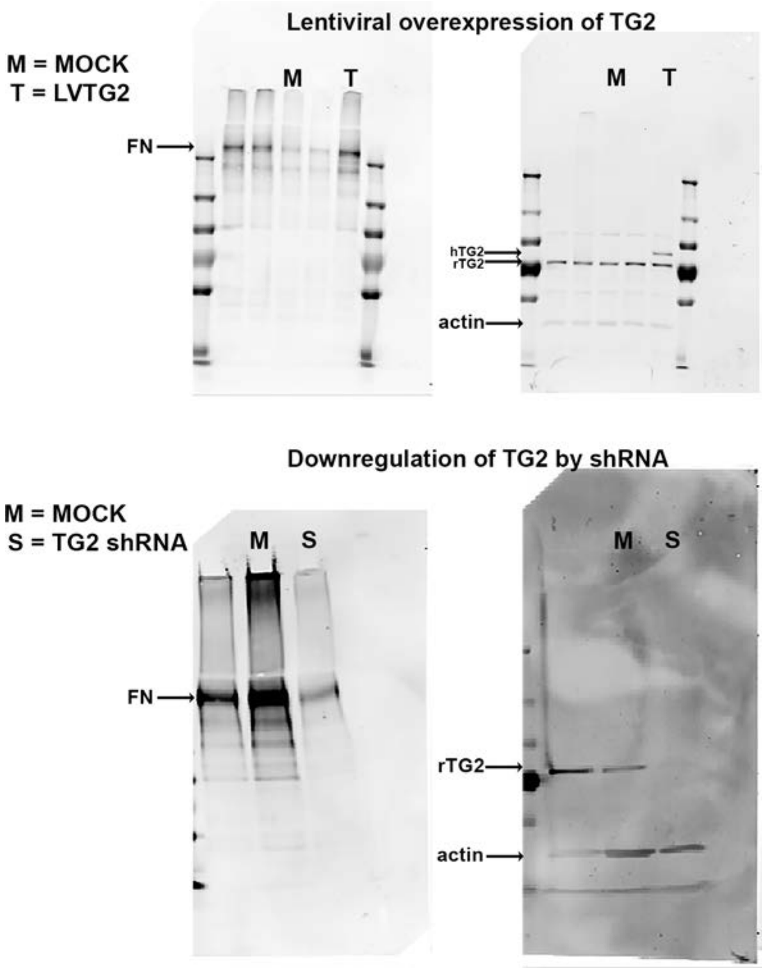

Figure S3. Full-length blots for figure 7. Top blots are from lentiviral overexpression of TG2. Lower blots are from down-regulation of TG2 by TG2 specific shRNA.
